# Microbial contamination of therapeutic contact lenses after photorefractive keratectomy: a prospective analysis

**DOI:** 10.1186/s12348-025-00469-7

**Published:** 2025-02-26

**Authors:** Mohammad Soleimani, Omid Behjati Najafabadi, Mehrnaz Atighehchian, Alireza Razavi, Zohreh Abedinifar, Seyed Ali Tabatabaei, Hassan Asadigandomani

**Affiliations:** 1 Department of Ophthalmology, The University of North Carolina at Chapel Hill, Chapel Hill, NC 27599 USA; 2 Ocular Trauma and Emergency Department, Farabi Eye Hospital, Tehran University of Medical Sciences, Tehran, Iran; 3 Research Center for Clinical Virology, Tehran University of Medical Sciences, Tehran, Iran; 4 Department of Pathology and Microbiology, Farabi Eye Hospital, Tehran University of Medical Sciences, Tehran, Iran

**Keywords:** Photorefractive Keratectomy, Contact lens, Microbial contamination, Refractive error, Microbial keratitis

## Abstract

**Purpose:**

The objective of this study was to examine bacterial contamination in therapeutic contact lenses (TCLs) utilized following photorefractive keratectomy (PRK) and to identify factors correlated to positive culture outcomes.

**Methods:**

This prospective study comprised 120 eyes from 60 patients who underwent bilateral PRK surgery at Farabi Eye Hospital in 2022. TCLs, applied postoperatively, were collected between the fifth and seventh days, placed in sterile containers with culture media, and analyzed for microbial growth. The documentation included patient demographic information, refractive status, preoperative conditions, culture results, and antibiotic susceptibility data.

**Results:**

The results indicated microbial growth was detected in seven lenses, which accounts for 5.8% of the total number of lenses (120 TCLs). Coagulase-negative staphylococci (CoNS) (4 lenses, 2 methicillin-resistant staphylococci (MRS)), Escherichia coli (E. coli) (1 lens), diphtheroid (1 lens), and micrococcus species (1 lens) were the isolated organisms. The patients did not experience any instances of microbial keratitis during the study period. Patients with positive cultures demonstrated a significantly higher mean age (35.00 ± 7.09 years, P-value = 0.036). No significant gender disparities were identified (P-value = 0.263).

**Conclusion:**

The incidence of microbial contamination in postoperative TCLs following PRK was minimal, with no occurrence of microbial keratitis. Older ages correlated with positive culture outcomes, highlighting the necessity for customized postoperative care approaches.

## Background

Surface ablation procedures have emerged as a pivotal advancement in the new century, captivating the attention of experts and enthusiasts alike [1, 2]. These techniques, known for their submicron precision, are commonly used for corneal surgeries such as photorefractive keratectomy (PRK), laser in situ keratomileusis (LASIK), and laser-assisted subepithelial keratectomy (LASEK) [3, 4]. PRK is a highly effective laser eye surgery to correct visual refractive errors, including myopia, hyperopia, and astigmatism [2]. Nonetheless, the potential for developing infections following PRK remains a concern [5–7]. During keratorefractive surgery, there is a potential for infection if the stromal surface or interface comes into contact with pathogens from the eyelids or contaminants in the operating room [8]. During the healing phase of PRK, the stroma stays exposed longer because of the epithelial defect caused by the procedure [9].

TCLs can lead to alterations in the tear film dynamics beneath the lens, resulting in diminished levels of antimicrobial components such as defensins, lysozyme, and lipocalins [10, 11]. This reduction in antimicrobial agents heightens the susceptibility to infectious keratitis, particularly when the lenses are worn during overnight sleep [12]. Although the risk of contamination exists, infections after keratorefractive surgery are uncommon [13, 14]. A previous study found 81.9% of pre-operative conjunctival swabs tested positive for bacteria. Staphylococci were present in 85% of the samples, followed by 4.8% gram-negative bacilli, 2.3% Staphylococcus aureus, and 1.2% Streptococcus pneumonia [15]. Feizi et al. observed that despite a 24.5% rate of microbial growth in cultures from the fornices before and after surgery, no corneal ulcers appeared [16].

It is crucial to evaluate the potential microbial contamination of TCLs utilized following PRK, despite the low risk of postoperative infection. These TCLs are generally employed to facilitate healing and safeguard the corneal surface; however, they may also serve as a reservoir for bacterial colonization during the epithelial recovery phase. In order to improve postoperative care, it is essential to understand the prevalence and types of microorganisms on TCLs, as well as the factors that influence contamination.

Advances in microbial detection (such as shotgun metagenomics) have helped to identify pathogens in ocular disease, therefore exposing often undetected bacteria and viruses by conventional cultures [17]. These methods offer important new perspectives on possible infection reservoirs, including TCLs applied following PRK. Additionally, systemic infections like SARS-CoV-2 may influence ocular microbiota, potentially affecting postoperative infection risks [18]. Including metagenomic techniques into postoperative treatment would improve early bacterial detection and direct focused antimicrobial treatments.

The purpose of this study was to investigate the microbial colonization of TCLs following PRK and its correlation with clinical factors in order to improve postoperative care.

## Methods

This prospective study involved 120 eyes from 60 patients who underwent bilateral PRK at Farabi Eye Hospital in Tehran, Iran, in 2022. The research adhered to the principles outlined in the Declaration of Helsinki and obtained formal approval from the institutional review board and ethics committee of Tehran university of medical sciences (IR.TUMS.FARABIH.REC.1400.083). All participants provided written informed consent.

### Eligibility criteria

Patients were eligible for inclusion if they were 18 years or older, had stable myopia or myopic astigmatism over the past 12 months, best-corrected visual acuity (BCVA) of 20/20 in each eye, and corneal topography with regular astigmatism. Patients with progressive or unstable myopia or astigmatism, active ocular diseases (e.g., glaucoma, uveitis, cataracts, blepharitis), a history of ocular surgery or trauma, the use of systemic medications affecting wound healing (e.g., corticosteroids, antimetabolites), or systemic conditions like diabetes, connective tissue disorders, immune deficiencies, or pregnancy were excluded. Moreover, individuals with dry eye, characterized by a tear breakup time (TBUT) of less than 10 seconds, as well as those with any history indicative of ocular infections, regardless of contact lens usage, were excluded. All patients were directed to utilize warm compresses and conduct eyelid care with baby shampoo for two weeks preceding the surgery. Moreover, individuals were instructed to refrain from touching their eyes while the TCLs were in place. Significantly, none of the patients displayed signs of active allergic conjunctivitis during the initial evaluations.

### Preoperative assessment

All patients received a thorough preoperative ophthalmologic assessment, which included uncorrected visual acuity (UCVA) and BCVA, manifest and cycloplegic refraction, intraocular pressure (IOP) measurement, slit-lamp biomicroscopy, dilated fundus examination, and corneal tomography utilizing Pentacam (Oculus Optigeräte, GmbH, Wetzlar, Germany). Soft contact lenses were discontinued a minimum of two weeks prior to the preoperative evaluation.

### Surgical procedure

All surgeries were performed in a sterile operating room environment. Topical tetracaine 0.5% was used for corneal anesthesia. After draping and preparing the eyelids with 10% povidone-iodine, a lid speculum was placed. The corneal epithelium was removed after applying 20% alcohol for 20 s. PRK ablation was performed using an excimer laser. Following the ablation, the cornea was irrigated with balanced salt solution. TCL was placed over the cornea using sterile forceps.

### Postoperative care

Postoperative treatment included chloramphenicol 0.5% eye drops every six hours for the first 10 days and betamethasone 0.1% eye drops every six hours for one month, followed by fluorometholone 0.1% (Fluocort) eye drops every eight hours for the subsequent three months. Patients were examined on the first and third postoperative day and between the fifth and seventh days, when TCLs were removed.

### Microbiological assessment

TCLs were extracted using sterile forceps and promptly placed into sterile tubes containing enriched thioglycolate liquid medium. The thioglycolate liquid medium was incubated at 37 °C for up to 14 days and monitored daily for microbial growth. Following the confirmation of microbial growth, cultures were transferred to blood and eosin methylene blue agar plates for the purpose of bacterial identification. Microbial identification relied on morphological, staining, and enzymatic activity criteria. Antibiotic susceptibility testing was conducted using the Kirby-Bauer disc diffusion method for all bacterial isolates except for Micrococcus and Diphtheroid species, for which the minimum inhibitory concentration (MIC) method was used to determine antimicrobial susceptibility. The selection of antibiotics for susceptibility testing followed Clinical and Laboratory Standards Institute (CLSI) guidelines. Quality control measures were implemented throughout the microbiological assessment process to minimize the risk of contamination and false-positive results. Plates were examined for potential contaminants, and strict aseptic techniques were followed during sample handling and processing.

### Statistical analysis

Continuous variables were tested for normality using the Kolmogorov-Smirnov test and presented as mean ± standard deviation (SD) if normal or median ± interquartile range (IQR) if non-normal. Qualitative data were reported as frequencies and percentages. The independent t-test was used to compare continuous variables between two groups, while the Chi-square test was applied for categorical variables. The likelihood ratio (LR) was calculated to assess the strength of relationships between variables, and Pearson’s correlation coefficient (r) measured correlations. Data analysis was conducted using SPSS version 26.0, with statistical significance set at P-value < 0.05.

## Results

A total of 120 eyes (60 patients; 41 females and 19 males) were enrolled in this study, with a mean age of 29.5 ± 7.3 years (ranging from 18 to 44 years). No significant difference was found between the ages of males and females (P-value = 0.34). Table 1 presents the clinical and demographic characteristics of the study participants (Table 1).

**Table 1:** Basic characteristics of study participants

Variables			Range	*P*-value
Age (year), Mean (SD)		29.5 ± 7.3	18 to 44	
Gender, *N* (%)	***Male***	19 (31.7)		0.34*
	***Female***	41 (68.3)		
Refractive error (Mean ± SD)	***Right Eye sphere***	-3.92 ± 1.15	-6.50 to -1.50	0.702**
	***Right Eye Astigmatism***	-2.23 ± 0.70	-6.00 to 0.00	
	***Left Eye sphere***	-3.73 ± 1.24	-6.75 to -1.50	
	***Left Eye Astigmatism***	-2.20 ± 0.72	-6.00 to 0.00	

*Chi-square **T-test

In this study, only seven patients tested positive in cultures. Four patients had cultures that showed coagulase-negative staphylococci (CoNS), two of which were methicillin-resistant staphylococci (MRS). One case identified Escherichia coli (E. coli), another case had diphtheroid, and one case revealed Micrococcus species. Among patients who had positive culture, only one patient was male and the mean of age was 35.00 ± 7.09 years. The average age of patients with a positive culture was significantly higher than that of patients with a negative culture (P-value = 0.036). There was no significant difference between the two patient groups regarding gender (P-value = 0.263). There was no significant difference in refractive error, including sphere and astigmatism, between patients with positive and negative cultures (P-values = 0.24 and 0.12, respectively) (Table 2).

**Table 2:** Demographic, clinical, and microbiological characteristics of patients with culture-positive results

Patient	Gender/Age (years)	Eye	Refractive Error (Sphere-Cylinder × Axis)	Microorganism
1	Male/39	Right	–1.00 − 1.25 × 175	MRS
2	Female/27	Right	–1.50 − 0.50 × 180	MRS
3	Female/33	Left	–1.50 − 0.75 × 10	Micrococcus
4	Female/41	Right	–1.50 − 0.75 × 140	Diphtheroid
5	Female/36	Left	–1.25 − 0.50 × 175	CoNS
6	Female/44	Left	–3.50 − 2.25 × 180	CoNS
7	Female/25	Right	–1.75 − 0.50 × 5	E. coli

**MRS;** Methicillin-resistant staphylococci, **CoNS;** Coagulase-negative staphylococci, **E. coli;** Escherichia coli

The antibiotic sensitivity profiles of the isolated microorganisms are presented in Table 3 (Table 3).

**Table 3:** Antibiotic resistance profiles of microorganisms isolated from contact lenses

Antibiotic	MRS	MRS	Micrococcus	Diphtheroid	CoNS	CoNS	E. coli
Oxacillin	R	R	-	R	S	R	-
Clindamycin	S	S	S	S	S	S	-
Erythromycin	S	S	S	S	S	R	-
Levofloxacin	-	-	S	S	S	S	S
Ofloxacin	S	R	-	-	S	R	S
Ciprofloxacin	S	R	-	R	S	R	S
Trimethoprim/sulfamethoxazole	S	R	S	-	S	S	-
Chloramphenicol	S	R	S	S	-	S	-
Ceftazidime	-	-	-	-	-	-	S
Amikacin	-	-	-	-	-	-	S

**S;** Sensitive, **R;** Resistant, **-;** Sensitivity test not done

## Discussion

In this study, cultures from seven TCLs following PRK tested positive for a range of bacterial strains, including CoNS, MRS, E. coli, diphtheroid, and Micrococcus species. The group with positive cultures was found to have a significantly higher mean age compared to those with negative cultures. However, no statistically significant difference was observed in the refractive error and gender between the two groups.

The identification of positive cultures in just seven patients revealed a diverse array of microorganisms. This finding emphasizes the variety of potential pathogens associated with postoperative contamination following PRK. CoNS, typically regarded as part of normal skin flora, have emerged as significant nosocomial pathogens, especially the MRS strains, complicating treatment and highlighting the need for stringent infection control measures [19]. The presence of E. coli and diphtheroids, although less frequently observed in ocular infections, suggests possible environmental contamination or patient-related factors contributing to colonization [20]. Furthermore, Micrococcus species, generally considered commensal organisms, can become pathogenic in immunocompromised individuals [21]. These findings underscore the importance of comprehensive preoperative screening and diligent postoperative monitoring to mitigate infection risks and improve clinical outcomes for patients undergoing PRK.

Our findings contrast with those of Pereira et al., who documented a positive culture rate of 16.7% in TCLs after PRK [22]. The reduced incidence observed in our study may be ascribed to variations in study populations, given that Pereira’s sample comprised hospital personnel, which could affect the contamination rate. The identified discrepancies underscore the necessity for customized infection control strategies and heightened vigilance in postoperative care, especially among populations with increased environmental exposure risks. Liu et al. observed higher bacterial colonization rates in TCLs after LASEK compared to PRK (16.7% vs. 6.67%, respectively). The contamination rate of TCLs post-PRK in Liu’s study corresponds with our findings; however, the increased rate following LASEK may be due to the epithelial flap created during the procedure, which fosters a favorable microenvironment for bacterial growth [23].

Despite 5.83% of TCLs testing positive for various microorganisms, there were no clinical infections reported among the patients. This supports the idea that infections are rare. It appears that several factors are more crucial in determining whether microbial colonization results in local infections, such as the application of topical antibiotics, the body’s innate immune response, the presence of antimicrobial proteins, and environmental conditions.

We found that the group with positive cultures had a significantly higher mean age than those with negative cultures indicates that age may significantly influence susceptibility to postoperative infections or bacterial colonization [24]. Several factors associated with aging could contribute to this increased risk, including a decline in immune system function, slower healing processes, and the presence of age-related comorbidities that may compromise overall health [25]. Moreover, older patients are likely to have a longer history of medication use or prior medical interventions, which could further impact their vulnerability to infections [26]. This data underscores the necessity of closely monitoring older patients following PRK and implementing targeted infection prevention strategies tailored for this demographic. It highlights the importance for healthcare providers to remain vigilant regarding the elevated risks faced by older patients and to consider additional prophylactic measures or enhanced follow-up care.

The microorganisms isolated in this study were consistent with those identified by Liu et al., including CoNS, MRS, Corynebacterium, and Micrococcus [23]. Our study also identified E. coli, which were not present in Liu’s dataset, indicating minor differences that may arise from variations in study settings or populations. Pereira et al. also reported Staphylococcus aureus and Streptococcus viridans as predominant isolates, with Bacillus species occasionally detected, further reflecting a diverse bacterial spectrum [22]. The predominance of Staphylococcus aureus in Pereira’s study may be attributed to occupational exposure in hospital settings, which contrasts with the similar microbial profiles observed in our study and Liu’s dataset [22, 23].

A comparative analysis of antibiotic sensitivity profiles demonstrates significant patterns across multiple studies. This study, similar to the findings of Liu et al., revealed a significant sensitivity of the CoNS to clindamycin, levofloxacin, and trimethoprim-sulfamethoxazole, underscoring the consistent efficacy of these antibiotics against methicillin-sensitive strains [23]. Micrococcus isolates in both Liu’s study and our research exhibited sensitivity to clindamycin and trimethoprim-sulfamethoxazole, with no resistance observed to other antibiotics [23]. Pereira’s findings on Staphylococcus aureus demonstrated universal antibiotic sensitivity, including to oxacillin and ciprofloxacin, which contrasts with the MRS noted in certain isolates in our study [22]. The lack of gram-negative organisms in Liu’s and Pereira’s datasets, in contrast to the presence of E. coli in ours, underscores the variability in microbial profiles across various clinical settings [22, 23]. The antibiotic sensitivity results across studies indicate a general efficacy of commonly used antibiotics; however, regional surveillance is essential to address emerging resistance patterns and optimize empiric therapy.

The findings of this study may improve clinical practice by highlighting the importance of mitigating microbial contamination following PRK, particularly in older patients who are more susceptible to bacterial colonization. Identifying various microorganisms and their antibiotic resistance profiles may improve more effective preventive and therapeutic methods. Furthermore, this data highlights the necessity for infection control surveillance, routine microbiological evaluations, and comprehensive assessment of antibiotic resistance trends regionally. However, further comprehensive multicenter investigations are necessary to establish more definitive recommendations for global application.

This study has several limitations that require attention. The restricted sample size of 60 patients (120 eyes) limits the statistical power and generalizability of the findings, especially given that only 7 cases were culture-positive, which diminishes the robustness of the results. Secondly, although the study was performed at a single institution, the findings may not apply to other settings with differing demographics, surgical techniques, or infection control protocols, thus requiring multicenter research to enhance generalizability. The exclusive use of thioglycolate liquid medium for culturing may have overlooked fastidious species, such as atypical bacteria or fungus, perhaps leading to an underestimation of contamination rates. Employing a wider range of culture techniques, including specialized media, may improve pathogen detection. Furthermore, confounding variables such as patient adherence to postoperative care, immunological condition, and environmental exposure may have influenced microbial colonization, highlighting the need for research that takes these factors into account. Additionally, while no patients exhibited active signs of allergic conjunctivitis during preoperative examination, the study did not account for a history of allergic conjunctivitis, which may have influenced ocular surface conditions and susceptibility to microbial colonization. The lack of a control group restricts our capacity to conclusively ascribe contamination rates only to the PRK process, as bacteria may potentially arise from pre-existing colonization or environmental exposure. Subsequent research using control groups, expanded sample sizes, and enhanced microbial detection techniques is crucial for validating our results and advancing the comprehension of infection risks following PRK. Notwithstanding these constraints, the work offers significant insights into microbial profiles post-PRK, highlighting the necessity for enhanced infection and postoperative surveillance.

## Conclusion

This study confirmed positive culture results in seven TCLs following PRK, indicating the presence of various bacterial pathogens, such as CoNS, MRS, E. coli, diphtheroids, and Micrococcus species. Notably, the patients with positive cultures were significantly older than those with negative cultures, suggesting that age may play a critical role in susceptibility. Future research should focus on larger sample sizes to further elucidate the factors influencing contamination risk, as well as to develop targeted interventions that enhance patient outcomes.

## Data Availability

Data supporting the findings of this study will be made available upon request from the corresponding author.

## Abbreviations

TCL: Therapeutic Contact Lens
PRK: Photorefractive Keratectomy
CoNS: Coagulase-Negative Staphylococci
MRS: Methicillin-Resistant Staphylococci
E. coli: Escherichia coli
LASIK: Laser In Situ Keratomileusis
LASEK: Laser-Assisted Subepithelial Keratectomy
BCVA: Best-Corrected Visual Acuity
UCVA: Uncorrected Visual Acuity
IOP: Intraocular Pressure
SD: Standard Deviation
IQR: Interquartile Range
LR: Likelihood Ratio
S: Sensitive
R: Resistant
